# Steered Molecular Dynamics Simulations of a Type IV Pilus Probe Initial Stages of a Force-Induced Conformational Transition

**DOI:** 10.1371/journal.pcbi.1003032

**Published:** 2013-04-11

**Authors:** Joseph L. Baker, Nicolas Biais, Florence Tama

**Affiliations:** 1 Department of Physics, University of Arizona, Tucson, Arizona, United States of America; 2 Department of Biological Sciences, Columbia University, New York, New York, United States of America; 3 Department of Chemistry and Biochemistry, University of Arizona, Tucson, Arizona, United States of America; Institut Pasteur, France

## Abstract

Type IV pili are long, protein filaments built from a repeating subunit that protrudes from the surface of a wide variety of infectious bacteria. They are implicated in a vast array of functions, ranging from bacterial motility to microcolony formation to infection. One of the most well-studied type IV filaments is the gonococcal type IV pilus (GC-T4P) from Neisseria gonorrhoeae, the causative agent of gonorrhea. Cryo-electron microscopy has been used to construct a model of this filament, offering insights into the structure of type IV pili. In addition, experiments have demonstrated that GC-T4P can withstand very large tension forces, and transition to a force-induced conformation. However, the details of force-generation, and the atomic-level characteristics of the force-induced conformation, are unknown. Here, steered molecular dynamics (SMD) simulation was used to exert a force *in silico* on an 18 subunit segment of GC-T4P to address questions regarding the nature of the interactions that lead to the extraordinary strength of bacterial pili. SMD simulations revealed that the buried pilin α1 domains maintain hydrophobic contacts with one another within the core of the filament, leading to GC-T4P's structural stability. At the filament surface, gaps between pilin globular head domains in both the native and pulled states provide water accessible routes between the external environment and the interior of the filament, allowing water to access the pilin α1 domains as reported for VC-T4P in deuterium exchange experiments. Results were also compared to the experimentally observed force-induced conformation. In particular, an exposed amino acid sequence in the experimentally stretched filament was also found to become exposed during the SMD simulations, suggesting that initial stages of the force induced transition are well captured. Furthermore, a second sequence was shown to be initially hidden in the native filament and became exposed upon stretching.

## Author Summary

There are a large number of infectious bacteria that can be harmful to humans. Some bacterial infections are facilitated by long, tether-like filaments called type IV pili which extend from the surface of bacterial cells and attach to the surface of host cells. Type IV pilus filaments can grow to be many micrometers in length (bacterial cells themselves, on average, are only a couple of micrometers in length and half a micrometer in diameter), and can exert very large forces (up to 100,000 times the bodyweight of the bacteria). Because they extend from the surface of the cell, type IV pili are very good candidates for drug targeting. Computer simulation was used to exert forces on a segment of one of these filaments, in an effort to mimic the effects of tension that would be experienced by the pilus upon binding during infection. Regions of the filament that become exposed to the external environment in the pulled state were determined, in an attempt to identify amino acid sequences that could act as targets for drug design.

## Introduction

Type IV pili (T4P), long (lengths at the micron scale) filamentous proteins composed of pilin subunits, are associated with a variety of bacteria, and emanate from the surface of the bacterial cell [1], [2]. T4P have been known as virulence factors for a long time as they are borne by many pathogens [1], [3]. They are of paramount importance in mediating attachment between bacteria and other surfaces, and perform a wide variety of functions for the bacterial cell including adhesion, motility, micro-colony formation, infection, and are implicated in immune escape [1], [3]. While other pili such as Type 1 or Type P pili provide function such as adhesion by their presence on the cell surface, T4P are also dynamic [4], [5]. T4P undergo cycles of elongation and retraction as pilin subunits are either added to or removed from the filament in a mechanism that is still poorly understood [1], [2]. When retracting, a single gonococcal (GC)-T4P filament can exert a force greater than 100 pN [6], [7]. The ability of GC-T4P to form bundles of 8–10 individual filaments has been observed, and these bundles can exert forces in the nanonewton range [8]. These are the highest recorded forces generated by bacteria (equivalent of 100,000 times the bacterial bodyweight).

Because of their involvement in surface attachment, GC-T4P filaments often find themselves under tension. The biological role of force in the interaction with host cells has been demonstrated to activate various mechanical signaling pathways in epithelial cells [9]. In addition, the physical forces exerted by the bacteria elicited dramatic rearrangements of the cell cortex [10], [11]. However, the mechanisms at play to go from force generation to biological function have yet to be established. Recent experimental evidence points to the impact of tension on the structure of T4P filaments. Specifically, experiments have shown conformational rearrangements of GC-T4P filaments expose buried amino acid sequences to the environment [12]. It is of interest to determine all of the regions exposed to the environment under tension for understanding the extraordinary plasticity of GC-T4P filaments. In addition, by uncovering what regions of the pilus filament become exposed under strain, more effective drugs, acting as inhibitors to T4P binding, could potentially be engineered [13], [14].

Among the many type IV pilins, the GC-pilin subunit, PilE [15], and the *Pseudomonas aeruginosa* subunit, PilA [16] have received the most attention, and their structures exemplify the canonical shape of type IV pilin: a globular head attached to a hydrophobic extended α-helix. In these two cases, the full-length subunits were crystallized. The N-terminal half of the helix (α1-N domain) protrudes from the protein, while the other half (α1-C domain) interacts with an anti-parallel four to five stranded β-sheet globular head domain. α1-N is almost completely hydrophobic except for a single charged residue, Glu5, which is conserved in nearly all type IV pilins, with only one exception: an aspartate is found at position 4 in the subunit PilS of *S. enterica* [1]. It has been speculated that Glu5 (and Asp4 in PilS), may serve to neutralize the electrostatic nature of the core of the filament by compensating for the positively charged N-terminus of α1-N [15], [17].

The globular head domain of the subunits lines the surface of the filament, and is therefore thought to be involved in its interactions with the environment [1]. The globular heads exhibit features relevant to pilus function. The αβ-loop possesses two post-translational modifications in GC-pilin, glycosylation of Ser63 and phosphorylation of Ser68 [1], [18], [19], which may protect epitopes from immune response and change the surface chemistry of the pilus and have been recently shown to play a role in the dispersal of the bacteria [18], [20], [21]. The D-region includes a hyper-variable loop, named as such because of the high variability of its amino acid sequence from one bacterial strain to another, which has been suggested to contribute to immune system evasion and persistent infection [22]–[24]. Additionally, along the filament surface, grooves between the globular heads of adjacent pilins are lined with positively charged residues in some locations, which may help to facilitate GC-T4P binding to DNA [15], [17].

**Figure 1:**
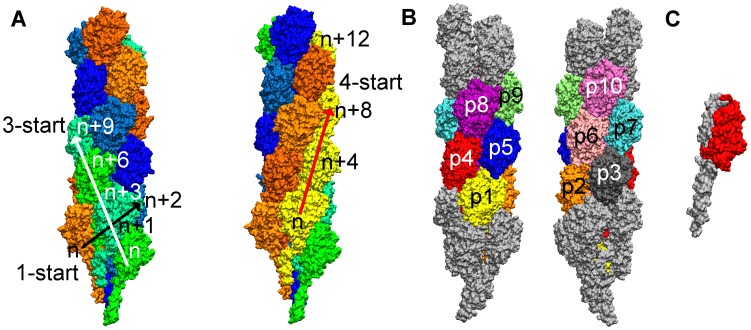
Filament labeling convention, depiction of pilin domains, and GC-T4P symmetries. (A) GC-T4P filament colored by 3-start helix (left) and 4-start helix (right). Black arrow indicates 1-start direction, white arrow indicates 3-start direction (left). Red arrow indicates 4-start direction (right). (B) GC-T4P filament. Pulled and fixed subunits (gray), “bulk” subunits (colored). (C) A single subunit colored by domain; the globular head domain (red) and α1 domain (gray).

A model for the GC-T4P filament assembly has been constructed by fitting the x-ray structure of GC-pilin (158 residues) into a cryo-EM map of a segment of GC-T4P filament at 12.5 Å resolution [17]. The cryo-EM reconstruction helped to shed light on its structural characteristics. Pilin subunits wind along the central filament axis, with approximately 3.6 pilin subunits per turn [17]. Subunits are arranged following symmetries along the filament axis (right-handed 1-start, left-handed 3-start and right-handed 4-start helices) [1], [2], [17]. These symmetries represent the various ways to divide the filament into progressions of pilin subunits that wind helically around the central filament axis. The right-handed 1-start helix symmetry describes positions of all pilin subunits in the filament using the smallest axial rise. The three left-handed 3-start helices of pilin subunits connect subunits n, n+3, n+6, etc, while the four right-handed 4-start helices of pilin subunits connect subunits n, n+4, n+8, etc, (see Figure 1A). The cryo-EM model also exhibited the presence of a channel of variable width (6–11 Å) through the filament core [17]. The α1-N domains of the subunits are thought to contribute to the strength of the filament due to their extensive hydrophobic interaction network in the core of the structure. Interaction between the N-terminal helices consists of about 75% of the total hydrophobic buried surface area of every pilin subunit [17]. While providing strength, the α1 helices are also expected to be flexible, since they possess glycine and proline that induce kinks and flexibility in α-helices. For example, Pro22 and Gly42 contribute to the S-like shape of α1 and are conserved amongst the Type IVa pilin [1]. In PilA from *Pseudomonas aeruginosa*, the conserved Pro22 residue leads to a kink in α1-N [16]. When over-expressed, PulG can form pilus filaments that have similar function to T4P [25]. If Pro22 is mutated in the pseudopilus PulG, pilus formation in *K. oxytoca* is significantly decreased, implying that the flexibility induced by Pro22 may be critically important for filament assembly [26]. The kink in α1-N has also been observed in recent crystal structures for the *D. nodosus* and *F. tularensis* pilins, with different crystallization procedures for the *F. tularensis* subunit implying that kinking in α1-N is natural [27].

Given the wealth of molecular data available on T4P and the importance of force in their biological roles, the powers of *in silico* methods were used to better understand the role of tension on T4P structure. MD simulations have been previously used to study filaments such as in the case of the actin filaments or microtubules [28], [29]. Complementing biophysical single protein pulling experiments, the effects of the application of external forces to proteins can also be studied using steered molecular dynamics (SMD) simulations [30], where external forces are applied to specific atoms in the biological system. Such SMD studies have been carried out on the adhesion protein FimH, a component of the related Type 1 pili [31], [32]. As T4P are known to sustain a considerable amount of force, understanding at the molecular level how T4P filaments respond under strain can provide insights into their function.

Therefore SMD simulations of GC-T4P using the 18 subunits long cryo-EM reconstruction were carried out to probe the dynamics of GC-T4P under tension, and to gain insights about the response of GC-T4P to external force at an atomistic level of detail. Even though the simulations are based on a 3-bundle model obtained from low-resolution cryo-EM experiments, predictions from simulation in agreement with experimental data would prove the possibility of such of model for the GC-T4P. The current study represents the first SMD simulation of a full pilus filament model, which would help contribute to the growing understanding of the wide variety of biological filaments found in nature. The aim of this computational study is to capture only the initial rearrangements of the filament coming under tension, as a full extension would be computationally prohibitive, in order to identify the strongest and weakest points of the filament structure. Structural changes in the GC-T4P filament, interactions between inter-subunit interfaces and residues that become exposed to the filament's external environment under tension, are discussed.

## Methods

### System preparation and simulation

The pdb coordinates for GC-T4P were obtained from the Protein Data Bank entry 2HIL [17]. It consists of 18 individual pilin subunits, each subunit being 158 residues in length (Figure 1B,C). This system was placed in a water box using the VMD [33] plug-in, Solvate (using the TIP3P force field for the water model), and waters within 2.4 Å of the protein were removed. The water box dimensions were ∼100 Å×100 Å×350 Å. The system was brought to electrostatic neutrality using the VMD Autoionize plug-in to add 47 Na^+^ ions and 29 Cl^−^ ions. Finally, additional water molecules within 2.7 Å of the alpha-helical core of the T4P system were removed to reduce the number of waters initially present in the filament core which is not expected to be filled with water [17]. This led to 287,272 atoms in the final system.

The package NAMD [34] was used with the CHARMM27 force fields [35] to carry out all simulations. Minimization was accomplished in two segments. First, 500 steps of minimization were carried out in which only the protein atoms were harmonically constrained, followed by 500 steps with all atoms in the system unconstrained. Subsequently to minimization, the system was equilibrated at a constant temperature of 310 K and a constant pressure of 1 atm with all atoms unconstrained for 500 ps. Constant temperature and pressure were maintained through the use of Langevin dynamics [36], with a Langevin damping coefficient of 5 ps^−1^ and a Langevin piston period of 0.1 ps, and periodic boundary conditions were used. The minimized and equilibrated structure served as the starting point for all simulations. A free simulation with no applied forces was carried out for an additional 20 ns with constant pressure and constant temperature maintained using the same parameters as in the 500 ps equilibration.

SMD simulations were carried out at constant velocity using the approach implemented in NAMD [34]. The spring constant was 500 pN/Å, and pulling velocities of 10 Å/ns, 5 Å/ns, 2.5 Å/ns, and 1 Å/ns were used. The four SMD simulations are referred to as T4P-v10, T4P-v5, T4P-v2.5, and T4P-v1 according to their pulling velocities. The SMD force was applied directly to the atoms in the top four subunits of T4P (Figure 1B) and the atoms in the first 30 residues of the four bottom-most subunits (Figure 1B) were fixed. Subunits that were not directly pulled on during the simulations are referred as the ‘bulk’ subunits. In all SMD simulations, pulling was stopped when the filament was stretched to the edge of its periodic box, which maintained a separation of approximately 30 Å between the filament and its periodic image along the z-dimension.

### Measuring lengths and distances

The length of a pilin subunit was defined as the distance between the center of mass of the alpha-carbon atoms for residues 1–3 and residues 51–53 for that subunit. The length of the complete GC-T4P filament was defined as the distance between the center of mass of the alpha-carbon atoms of residues 1–30 in each of the first four subunits (fixed selection) to the center of mass of the alpha-carbon atoms of residues 51 to 53 in the last four subunits (Figure 1B, subunits p7, p8, p9, p10) of the ‘bulk’ (i.e., excluding the four pulled subunits). Length extensions were defined as the difference between the GC-T4P length (or pilin subunit length) and its initial value.

The separations between globular heads were calculated as the distance between the center of mass of two subunit head domains. The distance between pilin subunits along the left-handed 3-start helices (subunit n, n+3, …) and the right-handed 4-start helices (subunit n, n+4, …) was calculated by finding the change in the z-coordinate of the center of mass of pairs of subunits (Figure 1A). The z-coordinate was used as the z-axis is approximately the central GC-T4P filament axis.

### Measuring angles

To measure bending angles in the N-terminal half of α1, each α1 helix was divided into three segments: residues 1–13, residues 15–21 and residues 23–53. The angle with Gly14 at its vertex was measured by defining a line of best fit for backbone atoms of residues 1–13 and residues 15–21. The angle made by these lines will be called θ_G_. Similarly for Pro22, the angle, θ_P_, was measured by calculating the angle made by the line of best fit for backbone atoms of residues 15–21 and residues 23–53, which have Pro22 as their vertex. 0 degree corresponds to a straight angle. A schematic of these angles can be viewed in Figure S1.

### Measuring contacts

Contacts were identified as existing between any two residues, which had any atoms coming within 3.3 Å of one another. The number of contacts was then monitored over time. Only the number of contacts based on proximity were tracked, and not their type. The contacts were monitored separately between the α1-domain interfaces, and for globular head interfaces.

### Averaging of results

For quantities that can be measured for each of the pilin subunits (for example, the tail extension, or the angles θ_G_ and θ_P_), data is presented as an average over the “bulk” subunits as defined above and also pictured in Figure 1B. For quantities which are measured for pairs of subunits (such as the separation between p3–p7, which is analogous by symmetry to the separation between p2–p6, p6–p10, etc.), an average over the value for all of the similar pairs is shown. For contacts between interfaces, a representative subunit from the filament, subunit p5 (Figure 1B) was chosen, with data from the T4P-v1 simulation mainly presented. The average number of contacts with all neighboring subunits whose interface involved the α1-domain were calculated. Similar calculations were performed for the contacts involving the globular head interfaces. Additional data for subunits p3, p4 and p6 are presented in the Supplemental Data, as well as data from simulations carried out at different pulling velocities.

### Solvent accessible surface area (SASA) calculations

The SASA for 5 amino acid long patches (5-mers) was calculated for each of ten frames over a period of 0.75 ns at the end of the T4P-v1 simulation that corresponds to an overall extension of the filament of 5%. The same criteria of 5% extension was also used to choose the frames over which the SASA calculation was performed for the other three SMD simulations. Similarly, SASA were calculated for the cryo-EM structure and over a period of 0.1 ns at the end of the free simulation. The final reported SASA values and their standard deviations were calculated by averaging over all ten frames, and then over the 10 “bulk” subunits (Figure 1B). SASA calculations were performed in VMD [33].

### Polyclonal antibodies

Polyclonal rabbit antibodies were raised against two regions of the pilin primary sequence around the regions that were thought to behave like SM1 in the molecular simulations (Genscript, Inc). Antibody #1 was raised against residues 94–108 (SSGVNNEIKGKKLSL) and antibody #2 was raised against residues 109–120 (WARRENGSVKWF). Those antibodies were further purified against bands of denatured pilins [37].

### Dot blots

Pili were purified as previously published [8]. 50 µL of purified pili in 50 mM CHES buffer (∼100 µg/mL) were either added to 50 µL of 50 mM CHES buffer or to 50 µL of 2X Laemmli buffer. The first solution was a solution of T4P filament, after 5 minutes boiling the second was a solution of pilin subunits (denatured pili). Dot blots of 2 µL of either solution were blotted twice on nitrocellulose membranes. The membranes were blocked with 5% dry milk in TBS for one hour, then incubated overnight with either antibody #1 or antibody #2 (1/1,000 dilution), washed 3 times with TBST, incubated for one hour with goat anti-rabbit HRP secondary antibodies (1/5,000 dilution) and revealed using ECL reagents.

### Molecular combing

Either unstretched or stretched (transitioned) T4P purified from *Neisseria gonorrhoeae* were obtain in a modified molecular combing technique [12]. Briefly pili sheared from *Neisseria gonorrhoeae* MS11 were first unspecifically labeled with carboxytetramethylrhodamine (TAMRA), a red fluorophore. They were then let to interact with clean coverslips for 15 minutes at the bottom of a 6 well plate (2 ml of the solution per well/coverslip). They were then either dried by removing excess liquid with a lint free Kimwipes tissue while maintaining the coverslip to obtain stretched samples or let as is to obtain unstretched samples. All wells were then fixed with 4% formaldehyde and subsequently processed for immunostaining.

## Results

A free simulation and four SMD simulations of the GC-T4P filament were carried out. The simulations were started from the equilibrated structure as described in the Methods section. As observations across all pulling velocities were similar, results for the T4P-v1 simulation are mainly described. The pulling velocity applied to this system is 1 Å/ns, which is the slowest that is computationally achievable in a reasonable amount of time, even though it is still several orders of magnitude faster than experimental speed [30].

### Filament conformational change

#### Filament length and diameter

**Figure 2:**
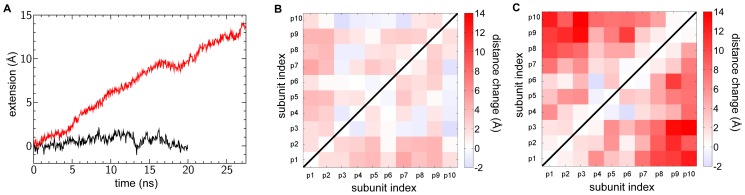
Extension of GC-T4P “bulk” versus time, and changes in separation between “bulk” subunit heads. (A) Extension of the GC-T4P filament versus time for the free simulation (black line) and T4P-v1 (red line). Distance change between the center of mass of “bulk” subunit heads between the end and the beginning of the (B) free simulation and (C) T4P-v1 simulation. Changes between subunit heads on the 1-start helix (p1–p2, p2–p3, etc.) are small, while large changes are observed between subunits along the 3-start and 4-start helices.

**Figure 3:**
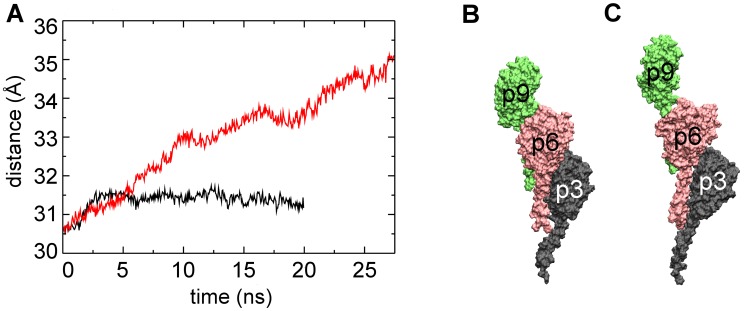
Average distance between pilin subunits along 3-start helical symmetry. (A) Average distance between the center of mass of bulk subunits along the three GC-T4P 3-start helices for the free simulation (black line) and T4P-v1 simulation (red line). Subunits from one of the 3-start helices from T4P-v1 (B) initial and (C) final frames. Coloring and labeling is the same as in Figure 1.

**Figure 4:**
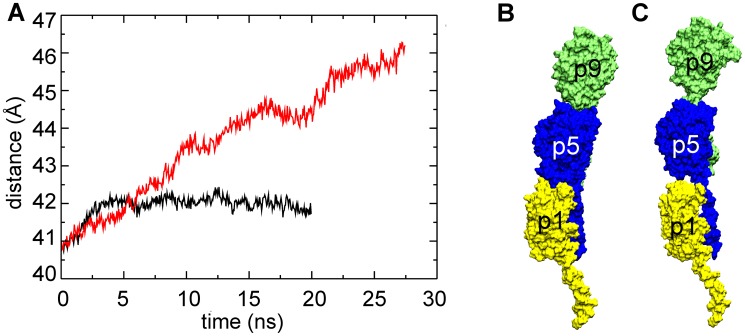
Average distance between pilin subunits along 4-start helical symmetry. (A) Average distance between the center of mass of bulk subunits along the four GC-T4P 4-start helices for the free simulation (black line) and T4P-v1 simulation (red line). Subunits from one of the 4-start helices from T4P-v1 (B) initial and (C) final frames. Coloring and labeling is the same as in Figure 1.

In the free simulation the length of the filament remained stable (Figure 2A). On the other hand in T4P-v1, an extension of 14 Å was observed for the ‘bulk’ subunits, from 156 Å to 170 Å, which resulted in several conformational changes within the filament. The extension due to tension promotes a more pronounced separation between the subunit heads than observed in the free simulation (Figure 2B,C). In particular, the axial separation between subunits in both the 3-start and the 4-start helices (see Figure 1A) increases over the course of the T4P-v1 simulation (Figure 3 and 4), while only small changes are observed between subunit heads on the 1-start helix (Figure 2C).

**Figure 5:**
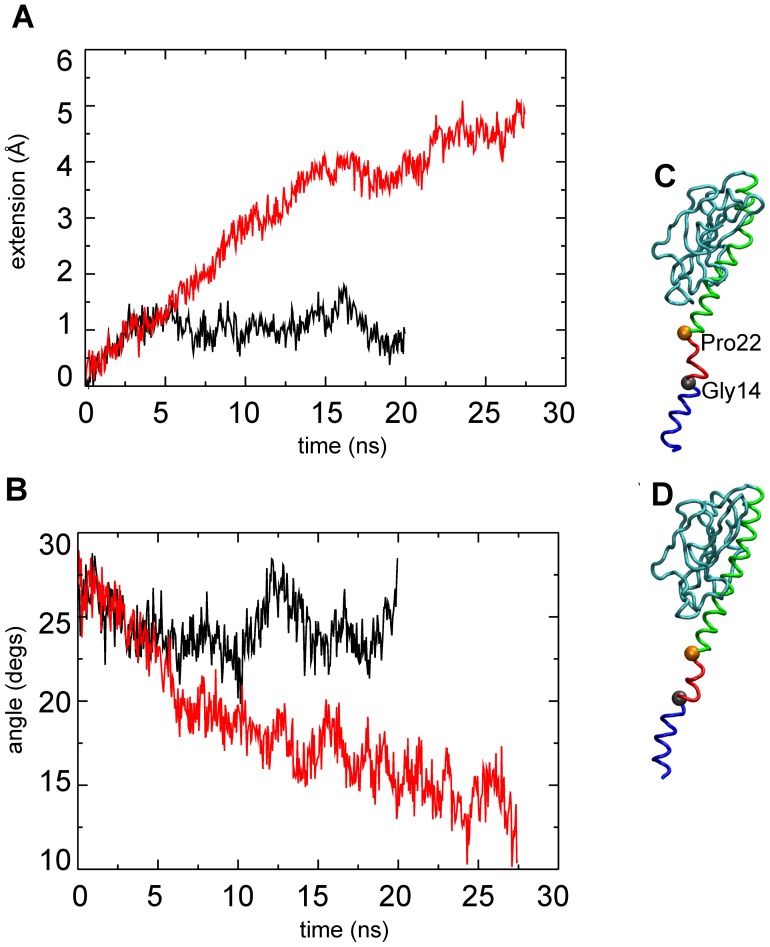
α1 domain extension versus time, and θ_G_ angle versus time for “bulk” subunits. (A) Average extension versus time for the α1 domains and (B) average θ_G_ angle. Free simulation (black line) and T4P-v1 (red line). (C) initial and (D) final snapshot of subunit p6 with residues 1–13 (blue), residues 15–21 (red), and residues 23–53 (green).

Individual pilin subunits become more elongated due to the straightening of the α1 domain (Figure 5A,B S1 and S2). Two residues in α1-N, Gly14 and Pro22, provide points of potential helical flexibility. Straighter helices are observed at the end of T4P-v1, as the angles θ_G_ and θ_P_ are reduced (Figure 5B,C,D and S1). While flexibility is observed in the free simulation, the degree of straightening is smaller. The considerable helical straightening observed upon pulling results in an average extension of the subunits of about 5 Å (Figure 5A). No significant rearrangements between the globular head and alpha-helical domains were observed during the simulation and the straightening of most helices is sufficient to explain the overall lengthening of the filament observed in the simulation. We note that the filament diameter remained roughly constant (Figure S3).

#### Central water channel

**Figure 6:**
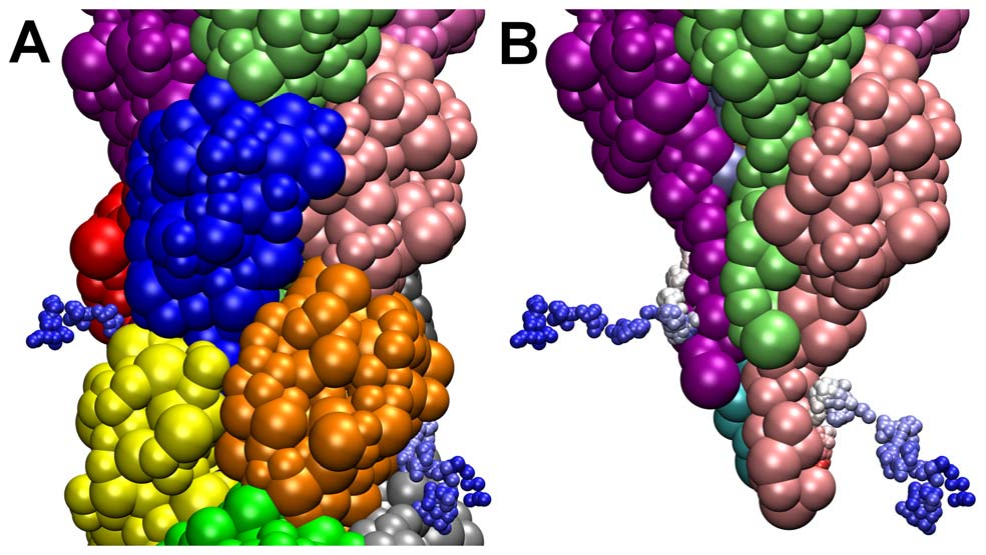
Diffusion of water from external environment to filament interior in the free simulation. Trajectories colored by time (from blue to red) of two water molecules (colored spheres) shown over the last 500 ps of the free simulation. (A) Water molecules can be seen moving from the exterior of the filament into the interior through gaps between globular heads on the pilus surface. (B) water molecules, upon entering the filament interior, interact with the α1-domain of pilin subunits (for clarity, some of the subunits shown in (A) are not displayed).

It has been proposed that a central channel of 6–11 Å diameter along the central axis of the filament could provide a compressible space allowing for pilus flexibility, and as such, it is not expected to be completely water filled [17]. To determine whether water molecules could exchange between the external environment and the interior of the filament, molecules that came within a close distance of the α1 domain of pilin subunits during a 500 ps window of the free simulation were first identified. Subsequently, these waters molecules were monitored to determine that they originally started from the exterior of the filament and not from base of the pilus which would be anchored into the bacterial membrane *in vivo*. We observed that these water molecules can exchange on a time scale of 500 ps. Two representative waters that diffuse into the filament to interact with pilin α1-domains are shown in Figure 6. Water exchange in the T4P-v1 simulation can also occur through larger gaps that appear on the filament surface as the globular heads separate from one another. These initial perturbations of the continuity of the filament might be the first steps towards the global reorganization of the pilus occurring experimentally but which cannot be captured here due to limited computational timescales.

### Contacts between subunit interfaces

To further characterize changes in the GC-T4P filament, subunit-subunit interfaces for four pilin subunits in the ‘bulk’ of the filament (subunits p3, p4, p5 and p6, see Figure 1B) were studied. Changes seen for ‘bulk’ subunits during the SMD simulations are expected to be more representative of what would occur in the GC-T4P filament in *in vitro* pulling experiments. Results for contacts for subunit p5 are presented here, while representative results for p3, p4 and p6 and for p5 at the pulling simulation T4P-v2.5 are presented in the Supplemental Data. Subunits p5 and p6 share 1-start, 3-start and 4-start interfaces only with other ‘bulk’ subunits; they do not share any interface with either fixed or pulled subunits. Subunits p3 and p4 share interfaces with fixed subunits as well as with ‘bulk’ subunits.

#### α1 domain

**Figure 7:**
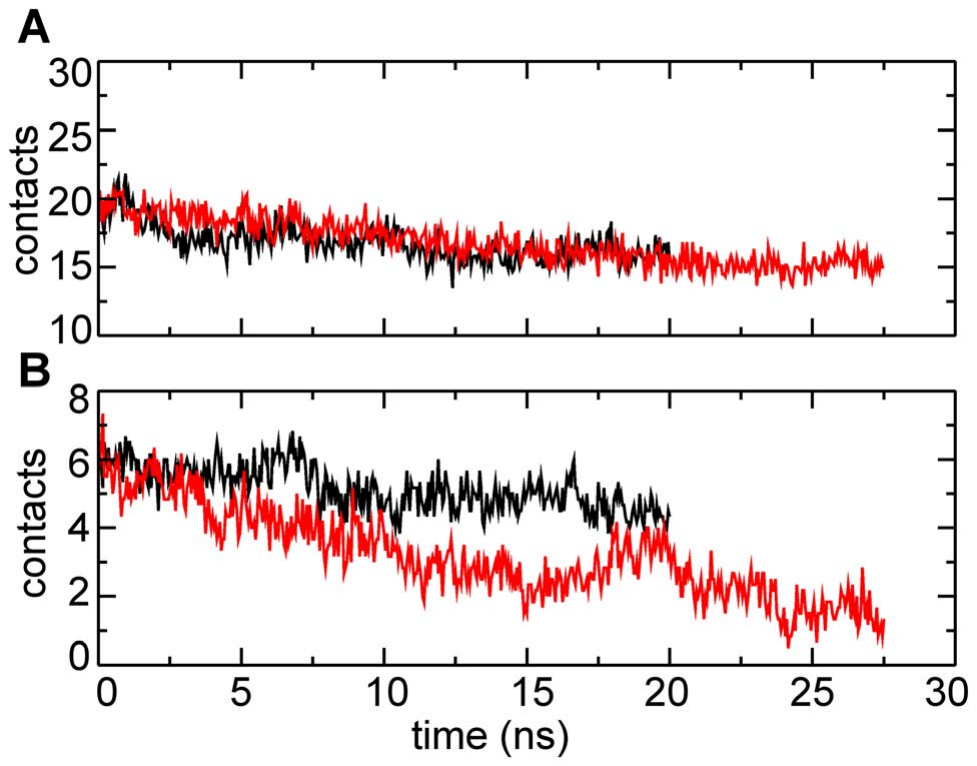
Average contacts between pilin domains for subunit p5 in free and T4P-v1 simulations. (A) α1-α1 contacts and (B) head-head contacts in the free (black lines) and T4P-v1 simulations (red lines).

Based on the model presented in [17], each pilin α1 domain has been proposed to make contact in helical ‘3-bundles’ with six neighboring α1 domains within the core of the GC-T4P filament, although there is no independent experimental evidence of such ‘3-bundle’ interactions. Nonetheless, our simulations probe the equilibrium state and pulled state of the filament under the assumption that the ‘3-bundle’ interactions are present in the intact filament. The α1-α1 contacts were well preserved compared to contacts between globular head domains in both the free and T4P-v1 simulations (Figure 7A,B for subunit p5) for all ‘bulk’ subunits (Figure S4) as well as for other pulling velocities (Figure S2). While many of these interactions are hydrophobic in nature, there is a conserved residue, Glu5, on each subunit in close proximity to the Phe1 nitrogen of the next subunit up the filament [1], [2], [17]. It is believed that the arrangement of these residues leads to interactions between the Glu5 side-chain oxygen and the Phe1 backbone nitrogen [17]. There is also evidence from the PAK pilin structure that the Glu5 side-chain oxygen can interact with the charged N-terminal residue Phe1 within a single pilin [16]. Measurements of hydrogen bonding between the Glu5 side-chain oxygen and the Phe1 nitrogen for both inter-subunit and intra-subunit interactions were performed. Both modes of contact (inter-subunit and intra-subunit) were observed in the free and T4P-v1 simulations. However, inter-subunit hydrogen bonding is more prevalent than intra-subunit hydrogen bonding (Table 1). Additionally, a larger number of inter-subunit Phe1/Glu5 hydrogen bonds were formed for a higher percentage of the simulation time in T4P-v1 than in the free simulation (Table 1).

**Table 1:** Number of subunits for which Glu5/Phe1 hydrogen bonding^1^ is observed.

	Free simulation		T4P-v1 simulation	
	intra-subunit^2^	inter-subunit^3^	intra-subunit^2^	inter-subunit^3^
**0<% simulation<25** 4	1	5	1	0
**25<% simulation** 5	2	3	1	7

1 Hydrogen bonds were defined with a 3 Å distance cutoff, and a 20 degree angle cutoff from linear bonding. Only hydrogen bonds involving “bulk” subunits were included in the analysis.

2 Intra-subunit Glu5/Phe1 hydrogen bonding occurs within a single subunit.

3 Inter-subunit Glu5/Phe1 hydrogen bonding occurs between the n to n+1 subunits in the GC-T4P filament.

4 Number of intra-subunit or inter-subunit Glu5/Phe1 hydrogen bonds that occur for less than 25% of the simulation time.

5 Number of intra-subunit or inter-subunit Glu5/Phe1 hydrogen bonds that occur for greater than 25% of the simulation time.

#### Globular head domain

Pilin subunit head domains make contact with other subunit heads along the 1-start, 3-start and 4-start helices. The 1-start interfaces (e.g., subunit p4, p5 and p6) are situated on the sides of the globular head domain, while the 3-start (e.g., subunits p2, p5 and p8) and 4-start (e.g. subunits p1, p5 and p9) interfaces are located at the top and bottom of the globular head domain (see Figure 1A and 1B). The cryo-EM structure shows interactions between the αβ-loop (residues 53 to 71) and the D-region (residues 121 to 151) of subunits along the 3-start helix, and interactions between the loops connecting β sheets for subunits along the 4-start helix [17].

Compared to the α1-α1 contacts that remain almost intact, a decrease of the number of contacts at the head-head interfaces was observed in T4P-v1 (e.g., Figure 7 and S4) as well as across additional pulling velocities (Figure S2). These interfaces correspond to contacts between globular head domains along the 3-start and 4-start helices. The decrease in contacts at these interfaces in T4P-v1 is consistent with the increase in the axial distance observed between pilin subunits as a result of SMD (Figure 3 and 4). Smaller decreases in the number of contacts between globular heads were observed in the free simulation (Figure 7 and S4). The interfaces along the 1-start helix have well-conserved contacts in both the free simulation and the T4P-v1 simulation (data not shown), which is consistent with the small separations between subunits along the 1-start helix (Figure 2).

#### Newly exposed epitopes

**Figure 8:**
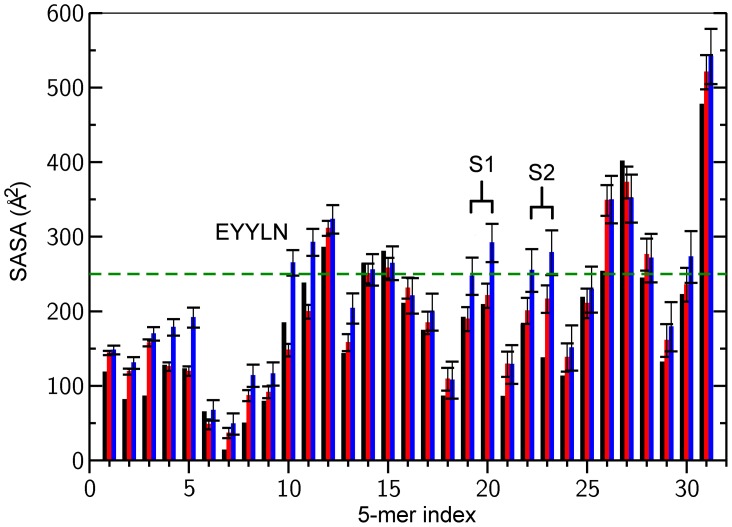
SASA for pilin 5-mers for cryo-EM, end of free simulation, and end of T4P-v1 simulation. SASA calculated for segments of 5 consecutive residues (5-mers) for the cryo-EM structure (black), end of the free simulation (red) and at the end of T4P-v1 (blue). Standard deviations are plotted as error bars. The green dashed line is set based on EYYLN's SASA in the pulling simulation, and represents the level used to predict other regions that might be hidden in the native filament, and exposed under tension. The first bin corresponds to residues 4 to 8. EYYLN index is 10. S1 (res 94–103) corresponds to indices 19/20 and S2 (res 109–118) to indices 22/23.

It was recently demonstrated that single GC-T4P under tension can transition to a totally different structure exposing epitopes previously buried [12]. The increased separations observed between the globular heads in the SMD simulations result in new regions of the filament becoming more accessible to the environment compared to the cryo-EM structure. In order to identify these newly exposed regions in the simulations, the average SASA for 5-mers across the pilin sequence for all subunits in the cryo-EM structure, the end of the free simulation, and at the end of the SMD simulations were measured (Figure 8 and S5). The underlying assumption is that the residues, which had a significant increase in SASA in the SMD simulation relative to the free simulation correlated with the epitopes that could become exposed upon force exertion.

**Figure 9:**
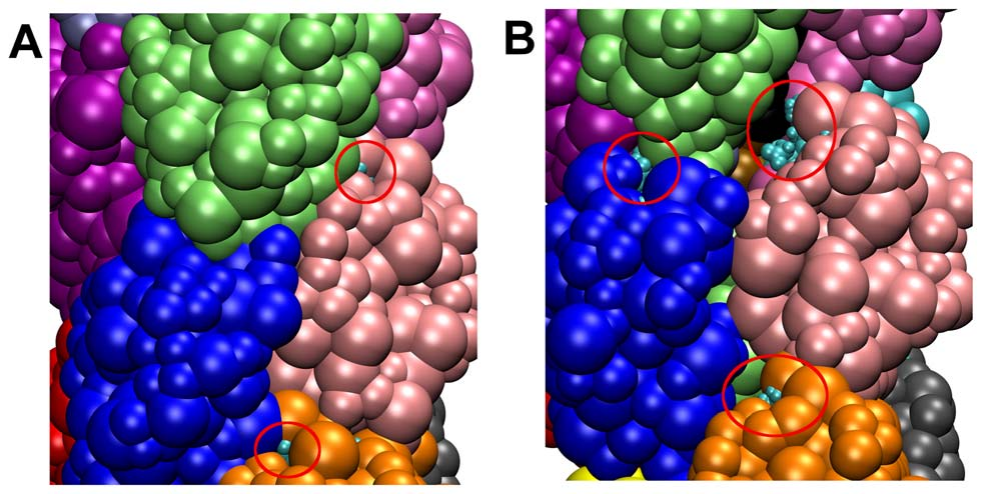
Exposure of EYYLN sequence in T4P-v1 simulation. EYYLN sequence (cyan spheres, circled in red) for three pilin subunits (dark blue, light pink, and orange) for (A) initial and (B) final snapshots from the T4P-v1 simulation. EYYLN becomes significantly more visible in (B) compared to (A).

The region with the largest SASA difference corresponded to the amino acid sequence EYYLN (residues 49–53), which has already been identified experimentally as becoming exposed upon stretching of the filament [12]. The EYYLN exposure in the simulations is due to the separation of the head-head interfaces creating gaps in the filament surface (Figure 9 and Movie S1). To predict other 5-mer sequences that might become exposed, regions with similar SASA increases to EYYLN (increase to ∼250 Å^2^ or greater) were identified (Figure 8 and S5). Two regions S1 (SSGVNNEIKG, residues 94–103) and S2 (WARRENGSVK, residues 109–118) met the criteria for increased exposure at various pulling velocities (Figure 8 and S5). At the end of three SMD simulations (T4P-v1, T4P-v5 and T4P-v10), the SASA for S1 (Figure 8 and S5) increased above the 250 Å^2^ threshold (e.g. bin 20) set based on EYYLN, while no substantial increase was observed in the free simulation. Similarly, upon pulling, S2 became more exposed in three simulations (T4P-v1, T4P-v10 and T4P-v2.5) (Figure 8 and S5, e.g. bin 23).

As the S1 and S2 sequences are less well hidden along the filament than EYYLN, dot blot and molecular combing experiments were performed to determine their degree of exposure in the native filament compared to the pilin subunits (Figure S6). Because these polypeptides were too small to elicit good immune response, antibodies against longer peptides were raised: antibody #1 against SSGVNNEIKGKKLSL (similar to S1 in Figure 8) and antibody #2 against WARRENGSVKWF (similar to S2 in Figure 8). These antibodies were tested to determine whether they would recognize pilin when assembled in pili or when denatured. Dot blot experiments reveal that antibody #2 recognizes its target peptide equally well, whereas antibody #1 shows a lack of recognition of pili in their native form (Figure S6A), consistent with previous studies [38]. Antibody #2 was already more exposed in the native form in our free simulation versus the cryo-EM structure (Figure 8), and as a longer peptide was used in experiments, it might explain recognition of pili in their native form. To further explore the binding pattern of antibody #1 to pili, T4P was subjected to molecular combing. Figure S6 B shows that unstretched pili do not show binding with antibody #1 whereas stretched pili do show binding of antibody #1, as predicted by our simulations.

## Discussion

### The three-helix bundles are proposed to anchor the core

The interactions in the core of the GC-T4P filament originate from the packing of the α1 domains against one another, and are thought to contribute to the incredible strength of bacterial pili. Each subunit's α1 domain has been proposed to make contact with the α1 domain of six other subunits by participating in three sets of ‘three-helix bundles’ based on the filament model describe by Craig et al. [17]. The SMD simulations demonstrate that such a model could allow for filament extension and underscore the strength of these non-covalent, and in many cases hydrophobic contacts between the α1 domains (Figure S7). Even as the filament and individual subunits extended in length (Figure 2 and 5), the contacts between α1 interfaces remained well conserved (Figure 7, S2 and S4), which have been suggested to provide stability to the filament [17], though such contacts might not be required for assembly, as it has been demonstrated that globular domains of the type IV pilins can assemble into fibers *in vitro* under certain sets of conditions [39]. These SMD simulations were performed to capture the initial steps of the elongation process, as elongations observed in experiments would be computationally prohibitive. Experimentally observed elongations would require an entirely new packing of the α1 domains to be realized. Coarse-grained simulations could provide insights to the nature of the packing in this extended conformation by probing numbers of contacts and residues in contact in the extended conformation.

One specific interaction, an inter-subunit Glu5 oxygen-Phe1 nitrogen hydrogen bond, is thought to be formed in order to neutralize charge in the filament core and increase hydrophobicity [17]. Additionally, in the crystal structure for the full PAK pilin filament a close contact in between the Phe1 backbone nitrogen and the Glu5 side-chain oxygen within a single subunit (intra-subunit interaction) was observed [16]. For the ‘bulk’ subunits inter-subunit hydrogen bonding between Glu5 and Phe1 was found to occur more frequently in the T4P-v1 simulation compared to the free simulation (Table 1), which could imply that the interaction also plays a role in maintaining stability in the core as the filament comes under tension.

### Flexibility of the α1 domain

Mutation of Pro22 in the pseudopilin PulG leads to a significant decrease in pilus formation in *K.* oxytoca, suggesting that the flexibility of α1-N around Pro22 may be critically important for pilus assembly [26]. Kinking of α1-N has also been observed in two more recent pilin crystal structures, suggesting that this bend is natural [27]. Additionally, it has been proposed that flexibility of the α1-N domain could lead to more efficient packing of pilin α1 helices within the filament core [16]. Fluctuations of angles θ_G_ and θ_P_ observed in the free simulation demonstrate the natural flexibility of α1, which could account for the effects observed upon assembly.

The observed elongations of the ‘bulk’ subunits (Figure 5) may represent initial stages of the transition to the force-induced conformation of GC-T4P that was recently observed experimentally [12]. The more extensive straightening of angles θ_G_ and θ_P_ in the T4P-v1 simulation may imply that the filament becomes less flexible as it is stretched, and that eventually all subunits in the pulled conformation become straightened.

### Stretched structure

In the experimentally determined stretched structure [12], the diameter of the filament decreases by 40% and its overall length increases by a factor of 3. In order to reproduce experimental data of this nature, the filament would need to be simulated for a much longer time and in a much larger water box along the z dimension (the filament axis), which would require simulations beyond the allowed time scale of all-atom MD. This computational study was designed to capture the initial rearrangements of the filament coming under tension, in order to identify the strongest and weakest point of the filament structure. While the actual extension of the filament involves a large increase in the axial rise per subunit, neither this feature nor a decrease in filament diameter (Figure S3) was captured by our simulations. However, elongations of α1 upon straightening of θ_G_ and θ_P_ could represent features in the initial stage of the transition towards the elongated GC-T4P conformation. Furthermore, the thinning of the filament observed experimentally may be the result of significant rearrangements of the pilin subunits that occur at timescales that these simulations cannot access.

The longer extensions of the filament observed experimentally would also require more extensive rearrangements of the pilin subunits than seen in the simulations. Conformational rearrangements between the globular head and the α1 domains of individual subunits are unlikely to produce longer extension; rather the slipping of α1 of one subunit along the α1 of adjacent subunits could produce such extension. In this case, neutralization of the charge of the Glu5 side-chain by hydrogen bonding to a residue on another subunit might become a concern, since of the first 23 residues of α1, only Glu5 is hydrophilic. However, residues 24 to 53 on α1 include side-chains available for hydrogen bonding or salt-bridge formation [17]. The α1 domain of a subunit could potentially slip out of its proposed 3-bundle interactions and translate up along the filament axis until Glu5 is able to interact with one of the adjacent subunit hydrophilic residues. To test this hypothesis, coarse-grained simulations would need to be carried out to study the filament at longer timescales. To further verify the models that would be obtained from such a computational approach, successful modeling of stretched pili based on cryo-EM would be useful, but such results are difficult to obtain.

### Motions of the head expose sequences for interactions

In contrast to α1, the globular heads of the pilin subunits are considerably more free to move. Water exchange between the external environment and the GC-T4P core can occur in spaces between the globular heads in the free simulation (Figure 6A), as well as through the larger gaps formed between globular heads due to the application of pulling forces. Waters that enter through the surface gaps can proceed to interact with the buried pilin α1-domains, which are water accessible even in the free simulation (Figure 6B).

In the functionally related, but structurally different Vibrio cholerae type IVb pilus (VC-T4P), deuterium exchange experiments demonstrated that the D-region (in the globular head domain) was significantly exposed, and hence could not be buried by interaction with the αβ-loop (connecting the α1 domain to the β-sheets), suggesting that the αβ-loop had to interact with another region of an adjacent subunit [40]. A recent cryo-EM study of VC-T4P shed further light onto the differences between GC-T4P and VC-T4P, including that VC-T4P packing is not as tight as the packing of subunits in GC-T4P, and that in VC-T4P a segment of the pilin α1 domains are exposed through gaps along the filament surface [41]. In the GC-T4P model, both the D-region and the αβ-loop are already well-exposed to the environment [17], [38], though some polar interactions are present [17]. Fluctuations observed in the free simulation can further diminish contacts between globular heads, which include the contacts between the D-region and the αβ-loop in GC-T4P, even when the filament is not under tension (see Figure 7, S2 and S4) as observed experimentally for VC-T4P. Reduction of contacts at these interfaces supports that the globular heads are not packed too tightly against one another, which would potentially limit filament flexibility [2], [17].

Experimentally, it has been observed that T4P can bundle, creating larger filaments able to exert greater force [8]. Pilus bundle formation might be occurring by initial binding of one T4P filament to a surface, which would result in its extension under tension, followed by the association of additional filaments to the initial one [8]. However, the mechanism by which subsequent filaments associate to the first filament is unknown. The increased space between globular heads observed in the SMD simulations, demonstrated both by increases in head-head distances (Figure 2) and changes in the 3-start and 4-start inter-subunit axial distances (Figure 3 and 4), potentially provide locations along the filament surface that adjacent filaments could ‘dock’ into, in turn promoting the creation of the experimentally observed T4P bundles.

Finally, the increased spacing between globular heads produced along the filament surface in the SMD simulations (Figure 2B,C for T4P-v1) also leads to the exposure of the EYYLN sequence (Figure 8, 9, S5 and Movie S1) and S1 (SSGVNNEIKG). The interest of predicting these regions of exposure lies in the possibility of understanding the plasticity of GC-T4P filaments and to potentially developing drugs that target T4P functions during infection. As these sequences were exposed further in the SMD simulations compared to the free simulation, it is most likely a direct consequence of the forces applied to the system. Exposure of EYYLN is consistent with the experimental result in [12] which showed EYYLN could bind with an antibody in its force-transitioned conformation, but not in the absence of tension forces. Exposure of SSGVN under force was demonstrated experimentally following prediction from our simulation.

Exposure of EYYLN and SSGVN in the SMD simulations suggests that a model based on the 3-helix bundle can capture conformational changes in the T4P filament that have been previously observed *in vitro* [12] or demonstrated in this study. Because simulations of the experimentally observed elongation would be computationally prohibitive, here only the initial changes were probed. *In vivo*, filaments are dynamic, constantly alternating between retraction and elongation phases while releasing some of the force they are subjected to. Therefore, our simulations also suggest that EYYLN and SSGVN might become accessible early on under physiological conditions.

## Conclusion

Conformational rearrangements of the GC-T4P filament under tension were studied utilizing MD simulations starting from the GC-T4P structure determined from a cryo-EM map and the crystal structure of a single GC pilin subunit. These studies were carried out in an effort to better understand the dynamics of the GC-T4P filament, its response to application of external forces and to probe initial stages of the transition between the relaxed and the tension-induced conformation.

Even though SMD simulations are based on 3-helix bundle model derived from low-resolution cryo-EM experiments, exposure of the sequences EYYLN and SSGVN, consistent with in-vitro experiments [12, present study], were observed. Therefore, such 3-helix bundle model could represent the actual structure of the filament. Simulations based on such a model reveal that the strength of the GC-T4P filament comes from the interactions between the α1 domains [17], as during elongation the contacts between these domains were well maintained. Contacts between subunit head domains decreased, creating additional gaps along the surface that could be related to filament bundling. These gaps lead to exposure of regions, which are hidden when not stretched, for potential drug targeting.

This work shows that SMD simulations can be used to narrow down the range of potential binding sites for drug therapy targeting bacterial filaments as the SSGVN was predicted as a possible site and confirmed experimentally. Finally, GC-T4P shares with the T4P from *Neisseria meningitidis* the presence of multiple post-translational modifications. As the functional importance of certain of these modifications is being discovered [21], simulations including the known modifications could shed more light on the function of the biological systems.

## Supporting Information

Figure S1 **Angle versus time (θ_P_) for “bulk” subunits.** (A) For each “bulk” subunit the angle θ_P_ is shown. Free simulation (black lines) and T4P-v1 (red lines). Graphs are labeled by subunit name from Fig. 1. (B) A schematic pilin subunit to depict the definitions of the angles θ_G_ and θ_P_.
(TIFF)

Figure S2 **Data for subunit p5 from the T4P-v2.5 simulation.** (A) Plot of θ_G_ versus time. (B) Extension versus time. (C) Average number of α1- α1 domain contacts versus time. (D) Average number of head-head contacts versus time.
(TIFF)

Figure S3 **Projection of backbone atoms onto x-y plane to depict filament diameter.** (A–F) 2-dimensional projections of backbone atoms x and y coordinates (excluding residues 1–53) for subunits colored in red in (G). Position of the atoms in the initial (black points) and final (red points) frame of the SMD and free simulations (A–E). (F) projection for the PDB structure.
(TIFF)

Figure S4 **α1- α1 domain contacts and head-head contacts for other “bulk” subunits.** The average number of α1- α1 contacts for subunits p3 (black), p4 (red) and p6 (green) as a function of time in the (A) free and (C) T4P-v1 simulations. The average number of head-head contacts for the same subunits (with the same coloring scheme) as a function of time in the (B) free and (D) T4P-v1 simulations.
(TIFF)

Figure S5 **SASA for pilin 5-mers for cryo-EM, end of free simulation, and end of T4P-v2.5/T4P-v5/T4P-v10 simulations.** SASA for 5-mers for the cryo-EM structure (black), end of the free simulation (red) and at the end of (A) T4P-v10, (B) T4P-v5 and (C) T4P-v2.5 (blue).
(TIFF)

Figure S6 **Dot blot and molecular combing results.** (A) Dot blots of assembled pili and denatured pilin. (B) Immunostaining image of stretched and unstretched pili. Antibody #1 and antibody #2 roughly correspond to predicted regions S1 and S2. In (B), the middle image in each set of 3 images is a merged image of the TAMRA result and the result from immunostaining with antibody #1.
(TIFF)

Figure S7 **Fraction of hydrophobic contacts at various p5 pilin interfaces in T4P-v1.** The number of hydrophobic contacts divided by the total number of contacts is shown for the interfaces between subunits p5 and p4 (black), p2 (red), p1 (green), p6 (blue), p8 (yellow) and p9 (brown).
(TIFF)

Movie S1 **Exposure of EYYLN in T4P-v1 simulation.** Pilin subunits are shown colored by subunit in a large bead representation. The four directly pulled subunits are not shown. The EYYLN residues are shown in a smaller sphere representation in cyan. First, a 360-degree rotation of the filament is performed to demonstrate that EYYLN is well-hidden around the pilus. The next segment shows the SMD simulation, and that gaps are exposed along the surface, which reveal EYYLN to the environment. Finally, another 360 degree rotation of the filament is carried out to demonstrate EYYLN exposure at the end of the SMD simulation around the entire pilus.
(MPG)
