# Supplementary figures and images for: Steered Molecular Dynamics Simulations of a Type IV Pilus Probe Initial Stages of a Force-Induced Conformational Transition
Source: PLoS Comput Biol. 2013 Apr 11;9(4):e1003032. doi: 10.1371/journal.pcbi.1003032 (PMC3623709; doi:10.1371/journal.pcbi.1003032)

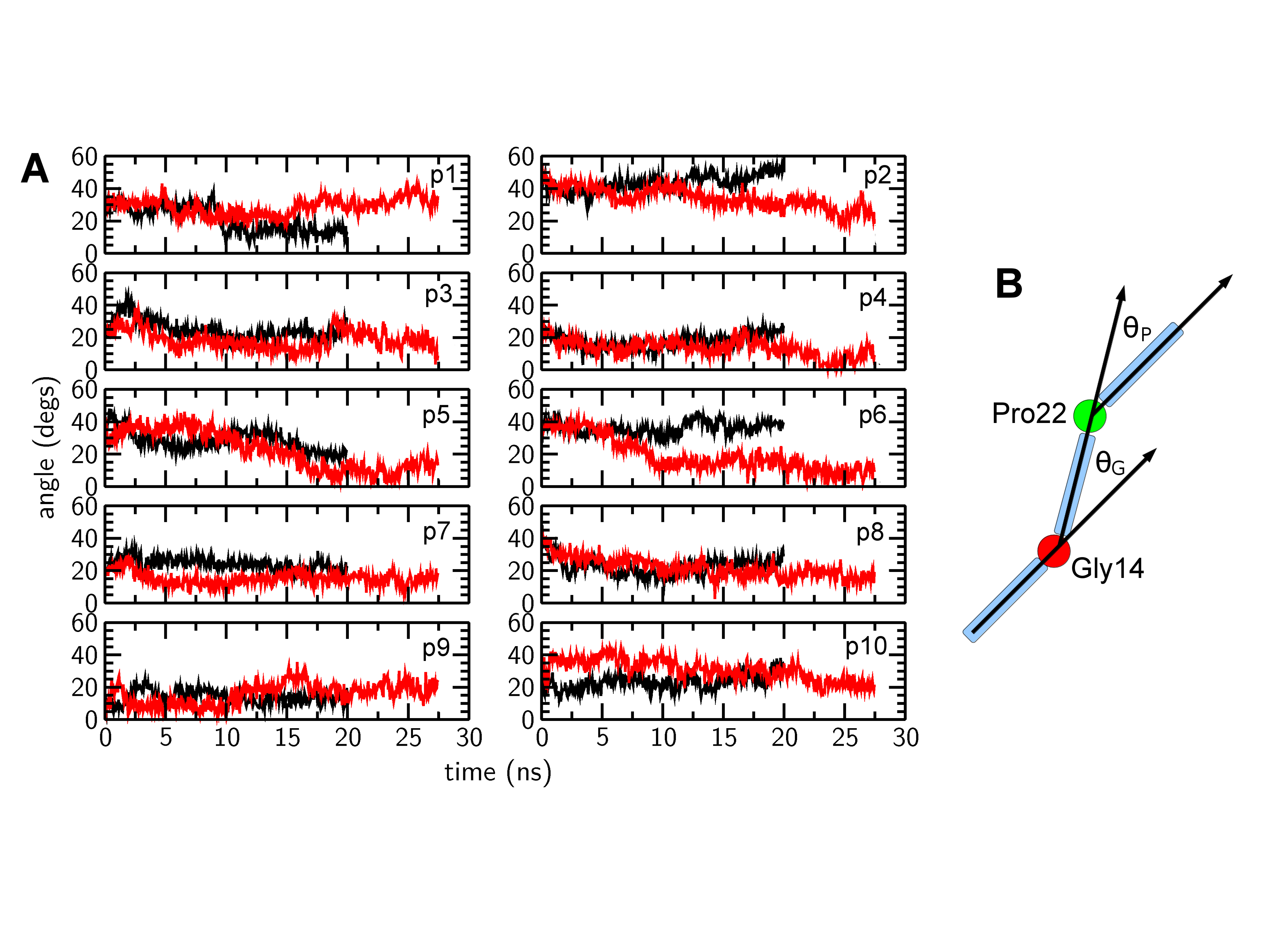

Supplement: Figure S1 — Angle versus time (θP) for “bulk” subunits. (A) For each “bulk” subunit the angle θP is shown. Free simulation (black lines) and T4P-v1 (red lines). Graphs are labeled by subunit name from Fig. 1. (B) A schematic pilin subunit to depict the definitions of the angles θG and θP. (TIFF) [file pcbi.1003032.s001.tiff]

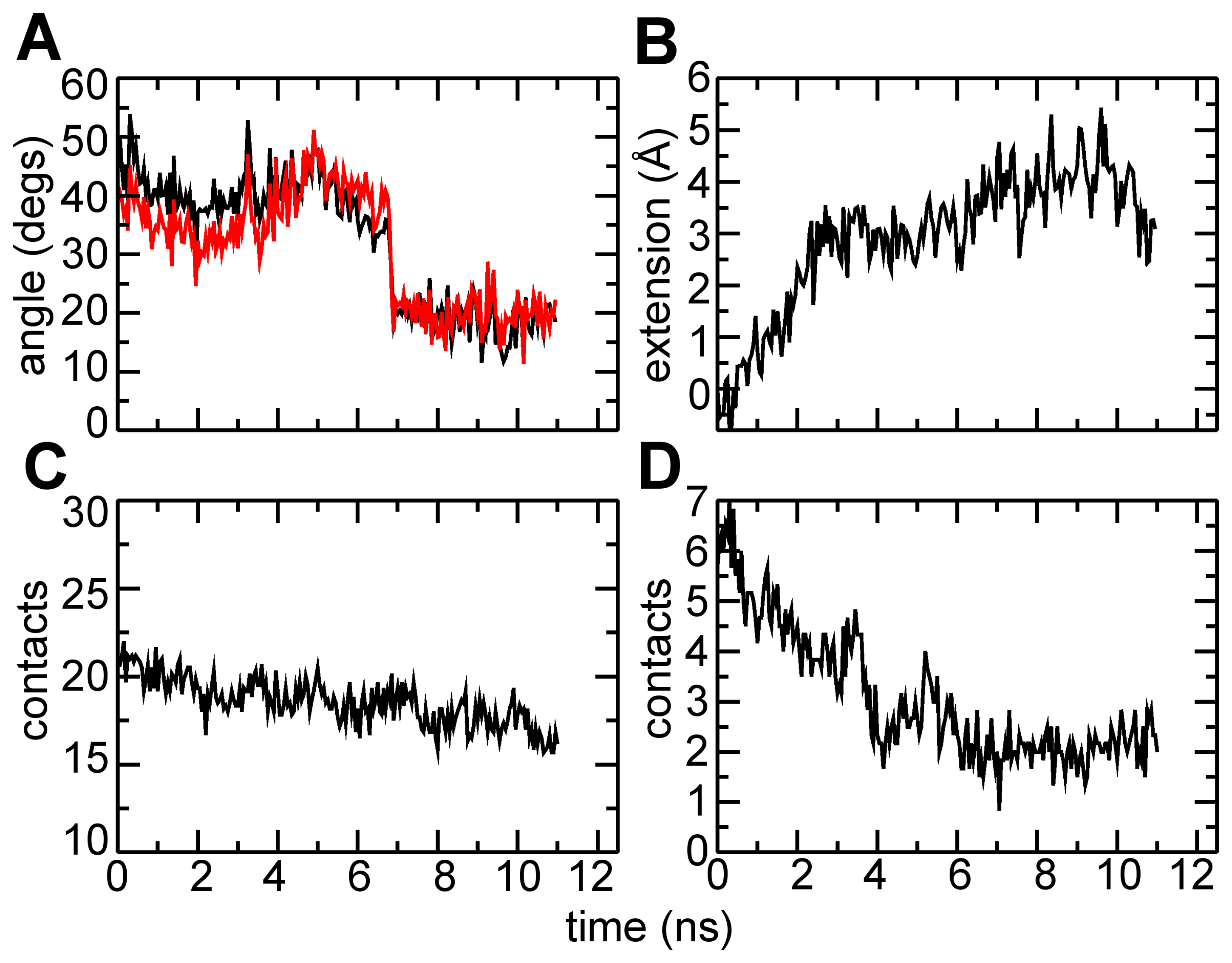

Supplement: Figure S2 — Data for subunit p5 from the T4P-v2.5 simulation. (A) Plot of θG versus time. (B) Extension versus time. (C) Average number of α1- α1 domain contacts versus time. (D) Average number of head-head contacts versus time. (TIFF) [file pcbi.1003032.s002.tiff]

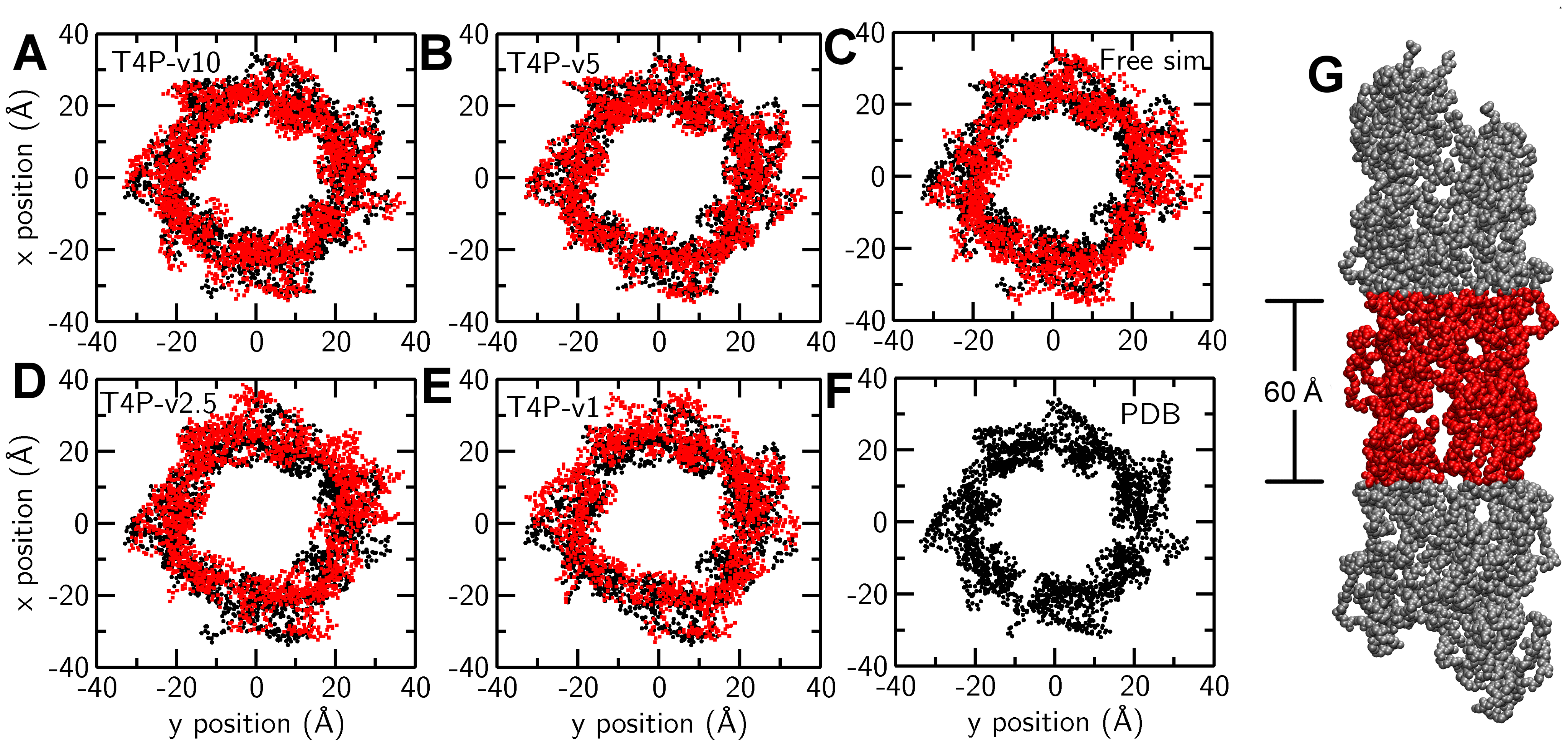

Supplement: Figure S3 — Projection of backbone atoms onto x-y plane to depict filament diameter. (A–F) 2-dimensional projections of backbone atoms x and y coordinates (excluding residues 1–53) for subunits colored in red in (G). Position of the atoms in the initial (black points) and final (red points) frame of the SMD and free simulations (A–E). (F) projection for the PDB structure. (TIFF) [file pcbi.1003032.s003.tiff]

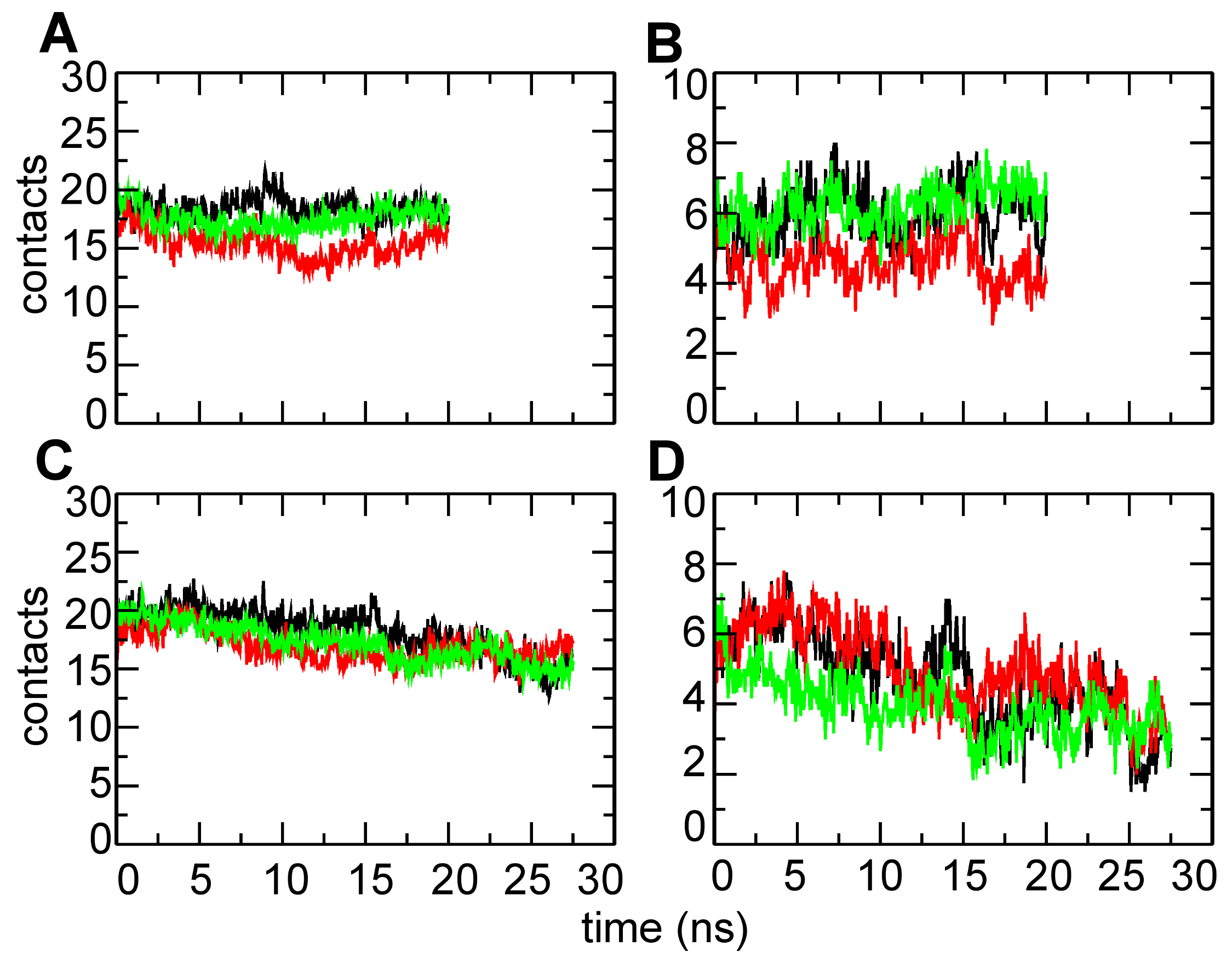

Supplement: Figure S4 — α1- α1 domain contacts and head-head contacts for other “bulk” subunits. The average number of α1- α1 contacts for subunits p3 (black), p4 (red) and p6 (green) as a function of time in the (A) free and (C) T4P-v1 simulations. The average number of head-head contacts for the same subunits (with the same coloring scheme) as a function of time in the (B) free and (D) T4P-v1 simulations. (TIFF) [file pcbi.1003032.s004.tiff]

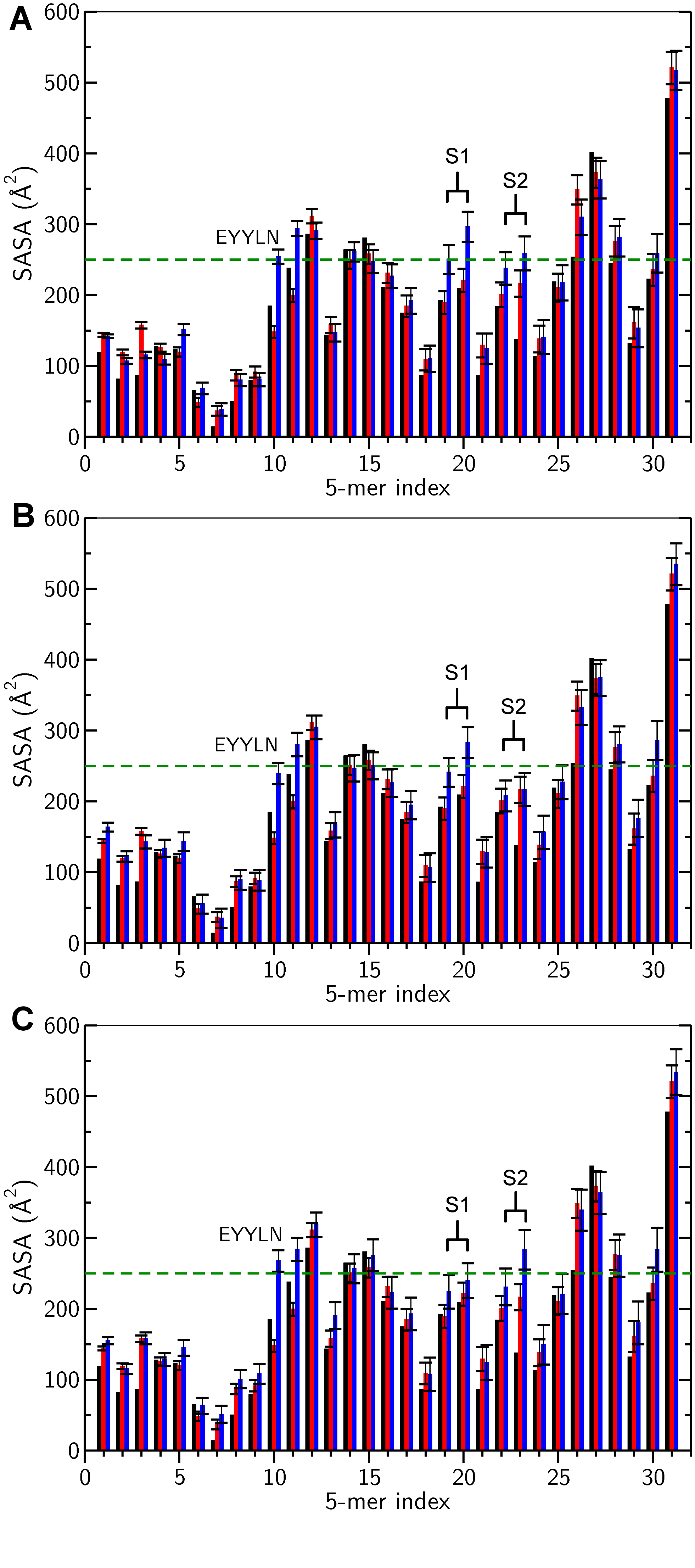

Supplement: Figure S5 — SASA for pilin 5-mers for cryo-EM, end of free simulation, and end of T4P-v2.5/T4P-v5/T4P-v10 simulations. SASA for 5-mers for the cryo-EM structure (black), end of the free simulation (red) and at the end of (A) T4P-v10, (B) T4P-v5 and (C) T4P-v2.5 (blue). (TIFF) [file pcbi.1003032.s005.tiff]

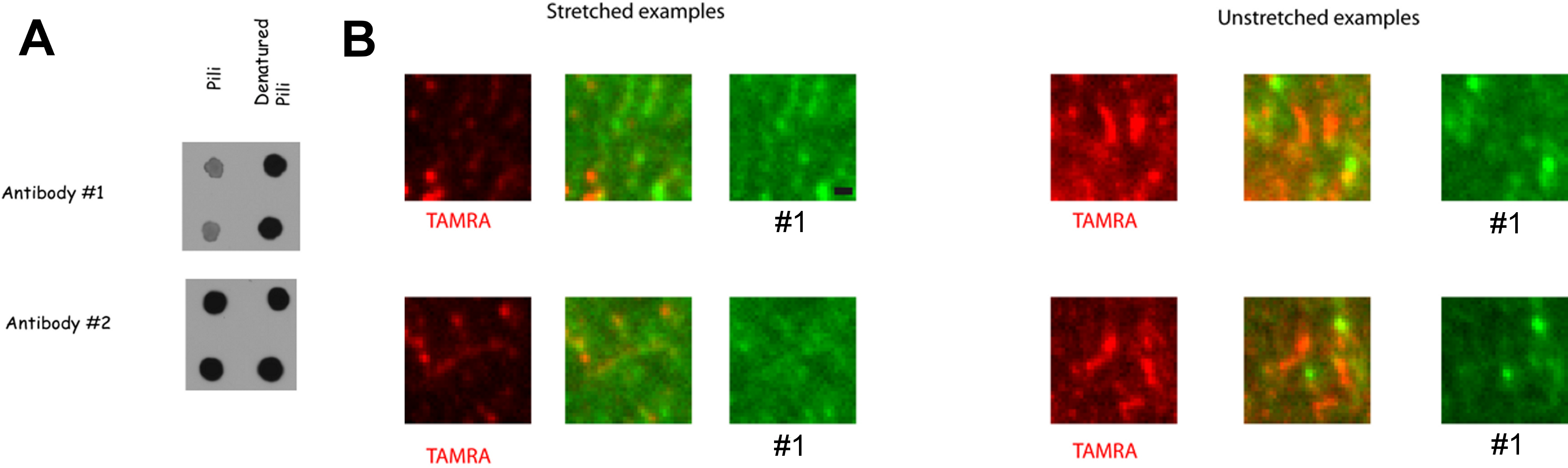

Supplement: Figure S6 — Dot blot and molecular combing results. (A) Dot blots of assembled pili and denatured pilin. (B) Immunostaining image of stretched and unstretched pili. Antibody #1 and antibody #2 roughly correspond to predicted regions S1 and S2. In (B), the middle image in each set of 3 images is a merged image of the TAMRA result and the result from immunostaining with antibody #1. (TIFF) [file pcbi.1003032.s006.tiff]

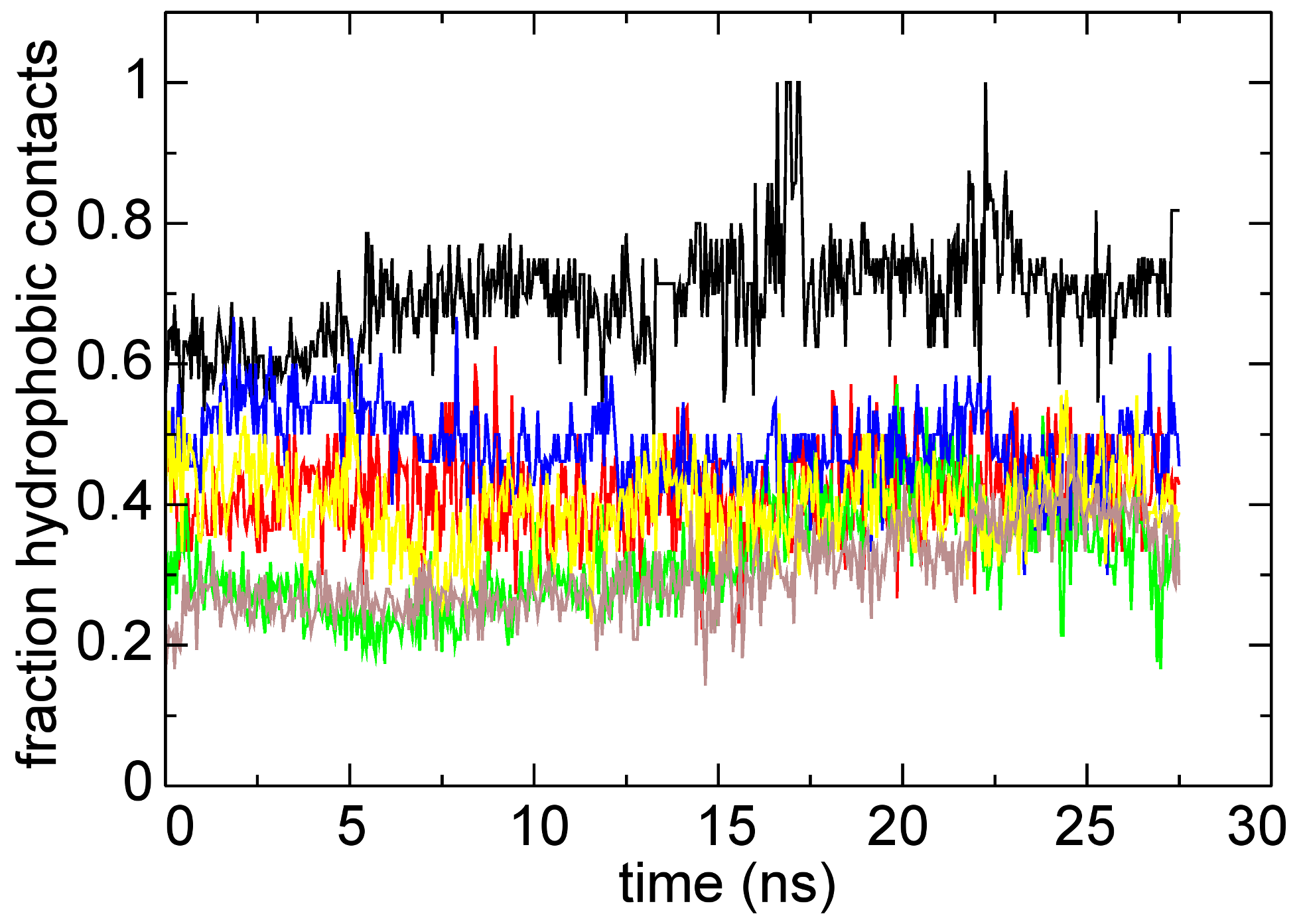

Supplement: Figure S7 — Fraction of hydrophobic contacts at various p5 pilin interfaces in T4P-v1. The number of hydrophobic contacts divided by the total number of contacts is shown for the interfaces between subunits p5 and p4 (black), p2 (red), p1 (green), p6 (blue), p8 (yellow) and p9 (brown). (TIFF) [file pcbi.1003032.s007.tiff]
